# Opposing Roles of pka and epac in the cAMP-Dependent Regulation of Schwann Cell Proliferation and Differentiation

**DOI:** 10.1371/journal.pone.0082354

**Published:** 2013-12-11

**Authors:** Ketty Bacallao, Paula V. Monje

**Affiliations:** 1 The Miami Project to Cure Paralysis, University of Miami Miller School of Medicine, Miami, Florida, United States of America; 2 Department of Neurological Surgery, University of Miami Miller School of Medicine, Miami, Florida, United States of America; Universidade Federal do Rio de Janeiro, Brazil

## Abstract

In Schwann cells (SCs), cyclic adenosine monophosphate (cAMP) not only induces differentiation into a myelinating SC-related phenotype, but also synergistically enhances the mitogenic action of growth factors such as neuregulin. To better understand the molecular mechanism by which cAMP exerts these apparently contradictory functions, we investigated the role of the two main effectors of cAMP, protein kinase A (PKA) and the exchange protein activated by cAMP (EPAC), on the proliferation and differentiation of both isolated and axon-related SCs. For these studies, a variety of PKA and EPAC agonists and antagonists were used, including pathway-selective analogs of cAMP and pharmacological inhibitors. Our studies indicated that the activity of PKA rather than EPAC was required for the adjuvant effect of cAMP on S-phase entry, whereas the activity of EPAC rather than PKA was required for SC differentiation and myelin formation. Even though selective EPAC activation had an overall anti-proliferative effect in SCs, it failed to drive the expression of Krox-20, a master regulator of myelination, and that of myelin-specific proteins and lipids, suggesting that EPAC activation was insufficient to drive a full differentiating response. Interestingly, inhibition of EPAC activity resulted in a drastic impairment of SC differentiation and myelin formation but not Krox-20 expression, which indicates an independent mechanism of Krox-20 regulation in response to cAMP. In conclusion, our data supports the idea that the outcome of cAMP signaling in SCs depends on the particular set of effectors activated. Whereas the mitogenic action of cAMP relies exclusively on PKA activity, the differentiating action of cAMP requires a PKA-independent (non-canonical) cAMP-specific pathway that is partially transduced by EPAC.

## Introduction

 The ubiquitous second messenger cyclic adenosine monophosphate (cAMP) is a key regulator of metabolic activity, survival, proliferation and differentiation in a wide variety of cell types. In particular, isolated cultured Schwann cells (SCs), the myelinating glia in the peripheral nervous system, are strongly dependent on the intracellular levels of cAMP. On one hand, cAMP is an instructive signal for cell cycle exit and differentiation into a phenotype that resembles that of the myelinating SC [1-3]. On the other hand and somehow paradoxically, cAMP is a strong mitogenic factor for SCs [4] and synergistically enhances cell proliferation in response to polypeptide growth factors that activate receptor tyrosine kinases (RTKs), such as PDGF and neuregulin [5-7]. In fact, it has long been recognized that in the absence of neurons, the proliferation of SCs in response to soluble neuregulin is relatively weak unless an agent that increases the intracellular levels of cAMP is added to the culture medium [8]. 

 In SCs, the transition from a proliferative (immature) to a differentiated (myelinating) stage is a developmentally regulated highly coordinated process that culminates with the production of a myelin sheath, a multispiraled extension of the plasma membrane that surrounds axons and allows the rapid conduction of electrical impulses. An early event in the process of differentiation is the upregulation of the transcription factor Krox-20/Egr-2 [9], a master regulator of myelination which drives the expression of an array of myelin-related proteins and lipids. These molecular changes occur in conjunction with the acquisition of a polarized and post-mitotic phenotype, the ensheathment of axons into one-to-one units and the wrapping of multiple layers of myelin membranes around higher caliber axons. Because of the strong pro-differentiating effects of cAMP observed in isolated SCs, it has long been suggested that a cAMP-dependent intracellular signal drives the process of myelination [1]. This concept has been supported, at least in part, by the dependence on cAMP of the expression of crucial regulators of the myelinating phenotype, including the transcriptional enhancers Oct-6 [10,11], Krox-20 [12] and NFκB [13] as well as the transcriptional inhibitor c-Jun/AP1, a negative regulator of myelination [14]. Yet, the signal transduction mechanism underlying the action of cAMP on the differentiation of myelinating SCs remains mostly undefined. 

 Accumulated evidence has indicated that cAMP controls complex cellular processes via changes in target gene transcription primarily through the activation of two downstream effectors, the cAMP-dependent protein kinase (PKA) and the newly discovered exchange protein activated by cAMP (EPAC) [15]. Upon binding of cAMP to the regulatory subunits, the catalytic subunits of PKA phosphorylate and modulate the activity of a variety of cytosolic and nuclear substrates, including the transcription factor CREB. On the contrary, EPAC directly transduces cAMP signals through its ability to act as a guanine nucleotide exchange factor for the small GTP-binding protein Rap1. Besides PKA and EPAC, other intracellular targets that bind cAMP through conserved cAMP-binding domains, including some cyclic nucleotide-gated channels, have been identified. However, their potential role in proliferation and differentiation is still elusive. 

 It has also became apparent that PKA and EPAC are able to simultaneously control multiple processes within the same cell and that the outcome of cAMP signaling may depend on the particular set of downstream effectors activated. Thus, we sought to investigate the differential contribution of PKA and EPAC to the cAMP-dependent regulation of SC proliferation and differentiation. To discriminate between the actions of PKA and EPAC, we used a variety of cAMP-stimulating agents, including pathway-selective cAMP analogs, along with pharmacological inhibitors to selectively interfere with PKA and EPAC signaling. Experiments were carried out using purified primary SCs growing in isolation (SC monocultures) and together with purified dorsal root ganglion (DRG) neurons (SC-neuron co-cultures). In turn, co-culture experiments were performed under conditions supportive of active cell division and myelin sheath formation, respectively. The side-by-side comparison of the mitogenic and differentiating responses of isolated and axon-related SCs allowed us to conclude that cAMP controls proliferation and myelination via different and independent downstream signaling mediators. Whereas the cAMP-dependent synergism of growth factor-induced proliferation relies on the activation of the canonical PKA pathway, differentiation and myelin formation are controlled non-canonically by cAMP through an EPAC-dependent mechanism. 

## Results

### Activation of PKA by forskolin was sufficient to synergistically increase the proliferation but not the differentiation of isolated SCs

 Forskolin, a direct and reversible activator of the transmembrane adenylyl cyclase (AC), has been used for many years as co-mitogen for isolated SCs due to its strong synergistic action on neuregulin-induced proliferation and negligible cytotoxicity [17]. Forskolin is a potent activator of PKA activity in SCs [23]. However, contrary to synthetic cAMP derivatives such as CPT-cAMP and db-cAMP, forskolin is a fairly weak inducer of SC differentiation even when provided at high concentrations [19,24]. Thus, we began these studies by comparing the potency of forskolin with that of cAMP analogs on the following cellular responses: 1) the induction of PKA and EPAC activities, 2) the synergistic enhancement of DNA synthesis in response to neuregulin, and 3) the expression of critical markers of differentiation into a myelinating SC-related phenotype. Proliferation assays evaluated the incorporation of [^3^H]-thymidine into nuclear DNA, a reliable measure of S-phase entry, in neuregulin-stimulated SCs. Differentiation assays evaluated the expression of markers typical of myelinating SCs, including the myelin proteins MAG and P_0_ and the myelin lipid galactocerebroside (cell surface O1) by means of immunofluorescence microscopy and western blot. We also evaluated the levels of expression and nuclear localization of the transcription factor Krox-20 along with the expression of immature/non-myelinating SC markers such as GFAP and c-Jun, which are both strongly reduced by treatment with cAMP [2,14]. Unless otherwise noted, experiments were done using primary SCs derived from adult rat sciatic nerves. 

**Figure 1:**
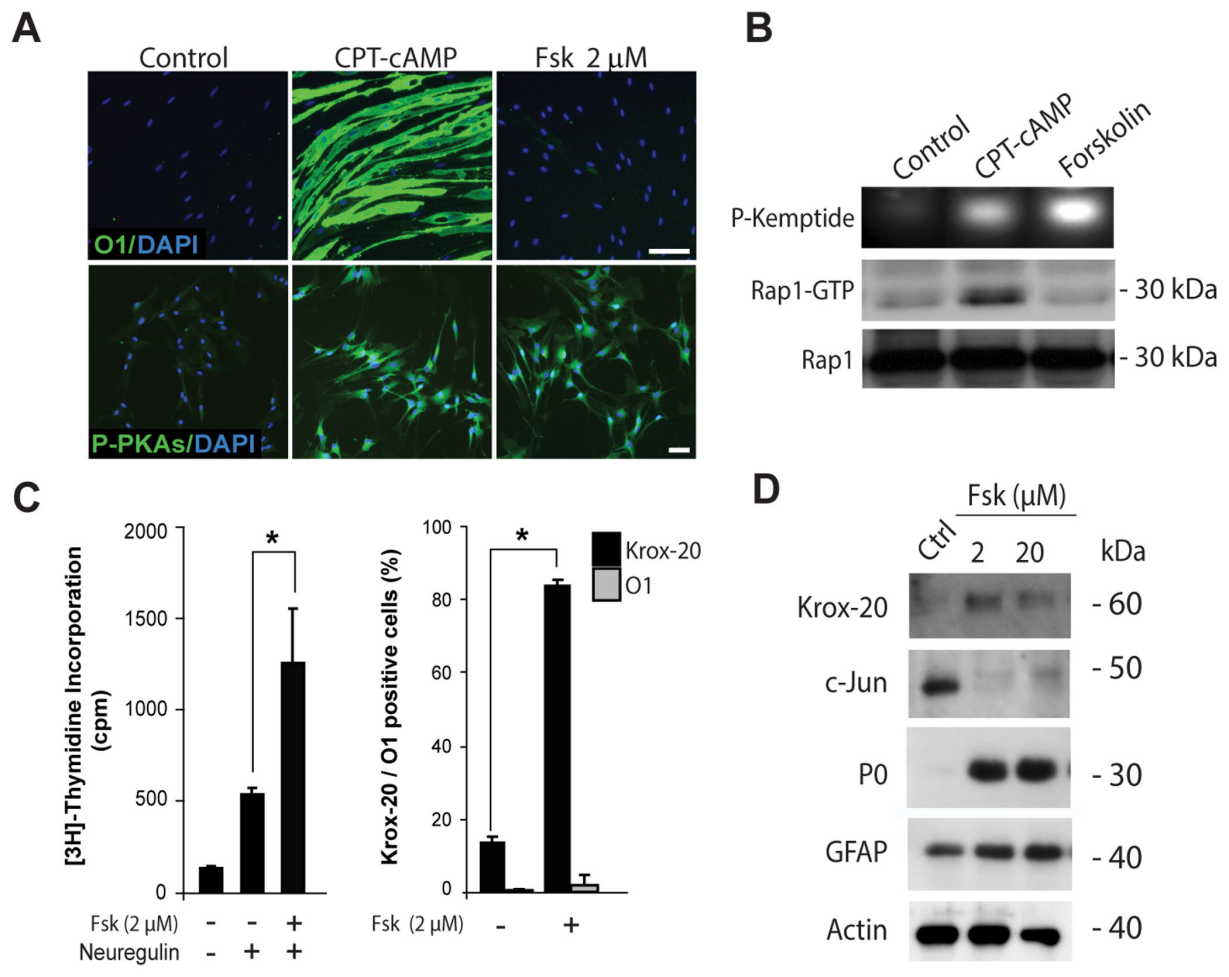
Effect of forskolin on the proliferation and differentiation of isolated SCs. Cultures of adult nerve-derived rat SCs were treated for 3 days with the indicated doses of forskolin (A-C), provided alone or together with neuregulin (10 nM). Cells were analyzed for the expression of SC differentiation markers by means of immunofluorescence microscopy (A, B, right panel) and western blot (B, D). Sibling cultures were also analyzed for the incorporation of [^3^H]-thymidine (C, left panel). The cell permeable analog of cAMP CPT-cAMP (250 µM) was used as a positive control for the induction of differentiation (A, O1 expression) and the dual activation of PKA and EPAC (B). PKA activity was assessed by the immuno-detection of phosphorylated PKA-specific substrates (A, P-PKAs) and the *in* *vitro* phosphorylation of the Kemptide substrate (B, P-Kemptide). EPAC activation was determined by pull-down assays of Rap1-GTP (B). PKA and EPAC activity assays used SCs stimulated with forskolin (2 µM) or CPT-cAMP (250 µM) for 30 min. The antibodies used are indicated in the figure. Nuclei were labeled with DAPI (blue). Results in all figures are representative of at least 3 experiments performed independently. In these and all subsequent graphs, bar heights are means of triplicate determinations; error bars represent S.D, and * represents statistical significance for p < 0.05. Scale bars correspond to 50 µm in all figures unless otherwise indicated.

 Results confirmed that similar to the action of cAMP derivatives, a mitogenic concentration of forskolin (2 µM) readily induced PKA activity in SCs, as assessed by the immunodetection of total phosphorylated PKA-specific substrates (Figure 1A, bottom panels) and the *in vitro* phosphorylation of a synthetic fluorescent peptide (Figure 1B, P-Kemptide). Along with the changes in PKA activity, forskolin synergistically enhanced neuregulin-induced SC proliferation (Figure 1C, left panel). However, as opposed to the action of cAMP derivatives, forskolin neither increased the activation of EPAC in SCs, as assessed by measuring the GTP binding activity of Rap1 (Figure 1B, Rap1-GTP), nor induced the expression of O1 (Figure 1A, upper panel, and 1C, right panel) and MAG (not shown). Yet, forskolin-treated SCs maintained their overall elongated morphology (not shown) and expressed high levels of GFAP (Figure 1D) despite exhibiting high levels of Krox-20 and P_0_ expression and negligible levels of c-Jun (Figure 1C-D). Results from additional experiments confirmed that forskolin alone failed to effectively induce differentiation (i.e. increase O1 and reduce GFAP expression) regardless of the use of higher concentrations (Figure 1D), prolonged incubation or repeated additions to the culture medium (not shown). 

 Collectively, these results suggest that PKA activation by forskolin effectively and selectively induced the proliferation rather than the differentiation of SCs growing in the absence of neurons. 

### The differentiation of isolated SCs was sensitive to the levels of intracellular cAMP rather than the activation of PKA

**Figure 2:**
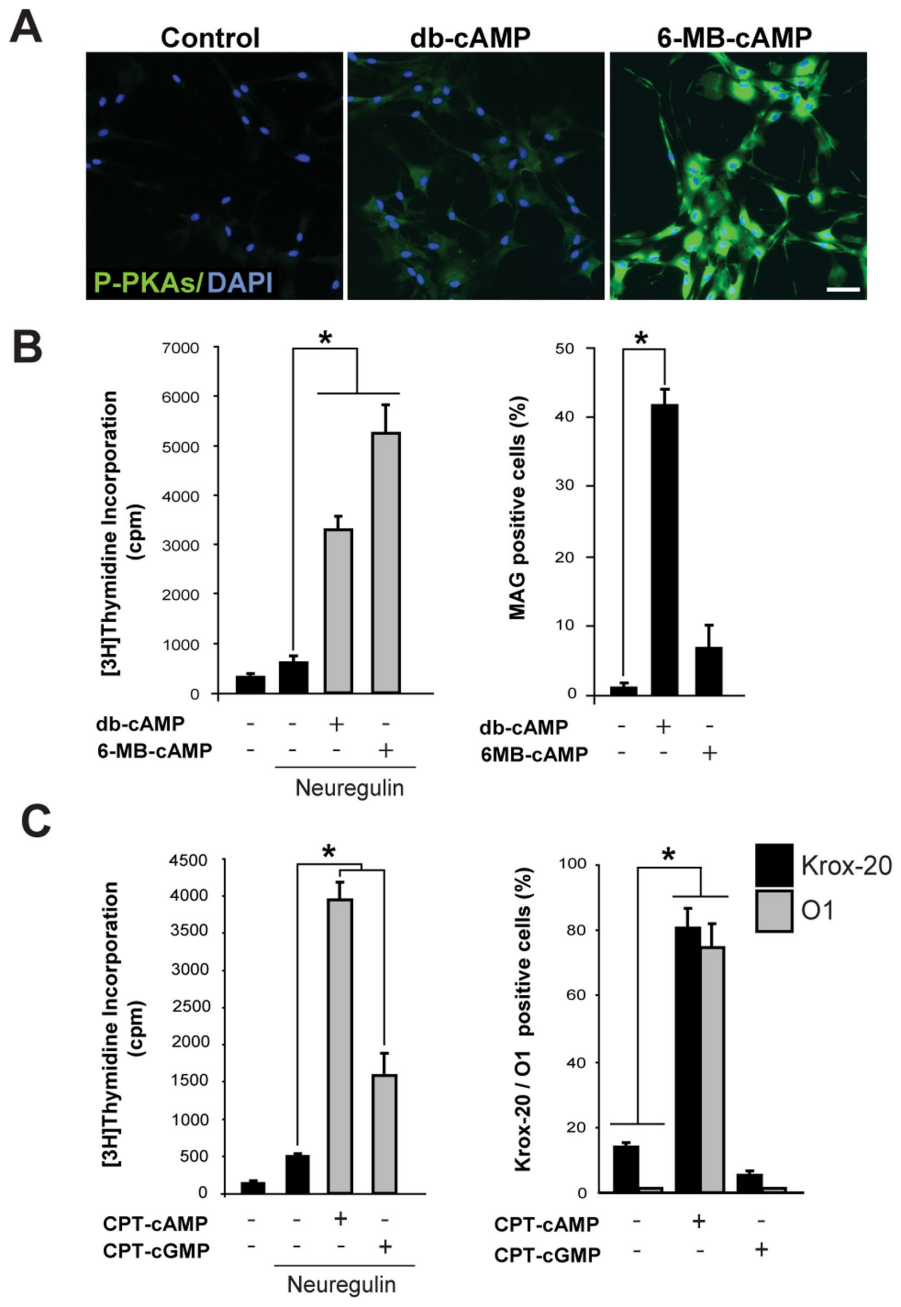
Effect of selective and non-selective activation of PKA on the proliferation and differentiation of isolated SCs. The assessment of PKA activity (A), S-phase entry (B-C, left panels) and differentiation (B-C, right panels) was done essentially as described in Figure 1. A quantitative data analysis of immunofluorescence microscopy images is presented in B, C (right panels). In A-B, the relative activities of non-selective (db-cAMP) and PKA-selective (6-MB-cAMP) analogs are compared in SCs stimulated for 30 min (A) and 3 days (B), respectively. db-cAMP and CPT-cAMP were used as positive controls for the induction of PKA activity (A), proliferation and differentiation (B-C). All analogs were used at a concentration of 200 µM.

 The above results also indicated that even though forskolin was sufficient to shift the balance Krox-20/c-Jun, an early cAMP-dependent event that occurs within the first 24 h post-stimulation [20], it was not sufficient to drive the expression of myelin proteins and lipids, which usually requires at least 3 days of persistent exposure to cAMP-stimulating agents [19,20]. The finding that PKA was the preferred substrate for forskolin-induced cAMP in SCs also indicated that the lack of differentiating activity could be due to the forskolin’s inability to activate EPAC signaling. This idea was supported by experiments using the PKA-selective analog of cAMP 6-MB-cAMP. This membrane permeable cAMP derivative has been widely used as a negative control for EPAC activation because it potently increases the activity of PKA without increasing that of EPAC [25]. Results showed that in isolated SCs, 6-MB-cAMP induced PKA activity and proliferation to levels significantly higher than those elicited by db-cAMP, as judged by the phoshorylation of PKA-specific substrates (Figure 2A) and the incorporation of [^3^H]-thymidine in the presence of neuregulin (Figure 2B), respectively. However, as opposed to db-cAMP, 6-MB-cAMP did not promote the expression of myelin-related proteins and lipids (Figure 2B, shown for MAG) or other features typical of cAMP-differentiated cells [19,26], such as a morphological transformation into a flattened epithelial-like shape (not shown). This result suggests that that induction of proliferation rather than differentiation directly correlates with the magnitude of the PKA-activating signal. 

 As a control for the specificity of action of cAMP analogs, we compared the mitogenic and differentiating activities of equivalent concentrations of CPT-derivatives of cAMP and cGMP, the latter being a closely related cyclic nucleotide as well as second messenger in mammalian cells. Results indicated that the induction of differentiation was highly specific to the action of cAMP, as the enhancement of neuregulin-induced DNA synthesis (Figure 2C, left panel) but not the expression of Krox-20 and O1 (Figure 2C, right panel) was at least in part mimicked by CPT-cGMP. 

 Altogether, these results support the idea that the differentiation of cultured SCs is a highly specific cAMP-dependent event that correlates with EPAC rather than PKA activity. 

### cAMP-induced SC proliferation but not differentiation was antagonized by pharmacological inhibitors of PKA

**Figure 3:**
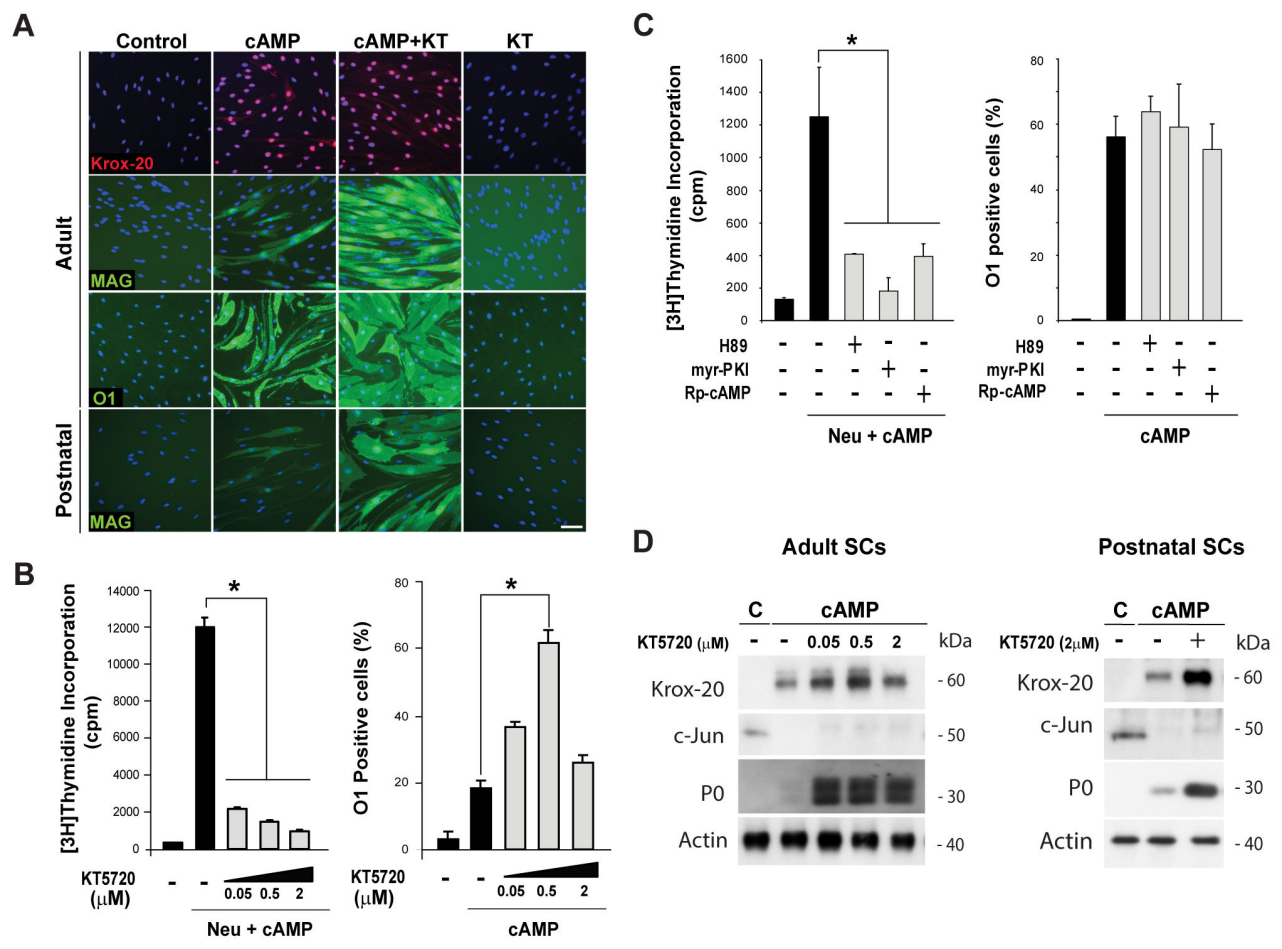
Non-antagonistic effect of PKA inhibitors on the cAMP-induced differentiation of isolated SCs. SC-only cultures were stimulated with CPT-cAMP (250 µM) provided alone (A-D) or in combination with 10 nM neuregulin (B-C) in the absence (control) or presence of PKA inhibitors (as indicated) for 3 days. Cells were evaluated for the expression of the indicated markers of differentiation by immunofluorescence microscopy (A-C) and western blot (D). In parallel, cultures were analyzed for the incorporation of [^3^H]-thymidine (B-C, left panels). In A and D, results were validated using cultures of postnatal rat SCs, as indicated. Inhibitors were used at the concentrations indicated in the figure or as follows: H89 (1 µM), KT5720 (1 µM), *myr*-PKI (40 µM), and *Rp*-cAMP (200 µM).

 Results using forskolin and PKA-selective cAMP analogs indicated that PKA activity was sufficient to enhance SC proliferation but not differentiation. Thus, we next analyzed whether PKA was required for the cAMP-induced expression of myelin-specific proteins and lipids. To begin addressing this question, we treated isolated SCs with increasing doses of KT5720, a well-known reversible inhibitor of the kinase activity of PKA, prior to cAMP administration and evaluation of the expression of markers of differentiation. As a control, we determined the effect of KT5720 on the incorporation of [^3^H]-thymidine, as G1-S progression in SCs is known to be antagonized by different pharmacological inhibitors of PKA [23,27]. To confirm the results, we performed a comprehensive study of the expression of markers typical of myelinating (e.g. Krox-20, O1, MAG and P_0_) and immature SCs (e.g. c-Jun) in cultures of adult and postnatal nerve-derived SCs. In turn, results were analyzed by means of immunofluorescence microscopy (Figure 3A-B) and western blot (Figure 3D). We found that KT5720 effectively and dose dependently blocked S-phase entry (Figure 3B, left panel) without antagonizing the effect of cAMP on differentiation (Figure 3A-B and D). Curiously, KT5720 dose dependently increased the expression of Krox-20, O1, MAG and P_0_ elicited by cAMP in both adult and postnatal SCs (Figure 3A, D) probably due to the well-known effect of this drug on promoting cell growth arrest [28]. Similar to KT5720, pre-incubation of SCs with H89 (a potent inhibitor of the kinase activity of PKA [29]), *Rp*-cAMP (the inactive stereoisomer of cAMP) and *myr*-PKI (a cell permeable myristoylated peptide from the heat-stable protein kinase inhibitor [30]) effectively antagonized the effect of cAMP on cell cycle progression but not differentiation (Figure 3C). Of note, persistent administration of the above mentioned PKA inhibitors did not induce differentiation in the absence of cAMP (Figure 3A, shown only for KT5720). 

 Contrary to the PKA-dependent regulation of proliferation, these results clearly suggest that PKA activation in SCs is for the most part dispensable for the pro-differentiating action of cAMP. 

### Axon contact-induced SC proliferation but not the formation of myelin sheaths was antagonized by PKA inhibitors

**Figure 4:**
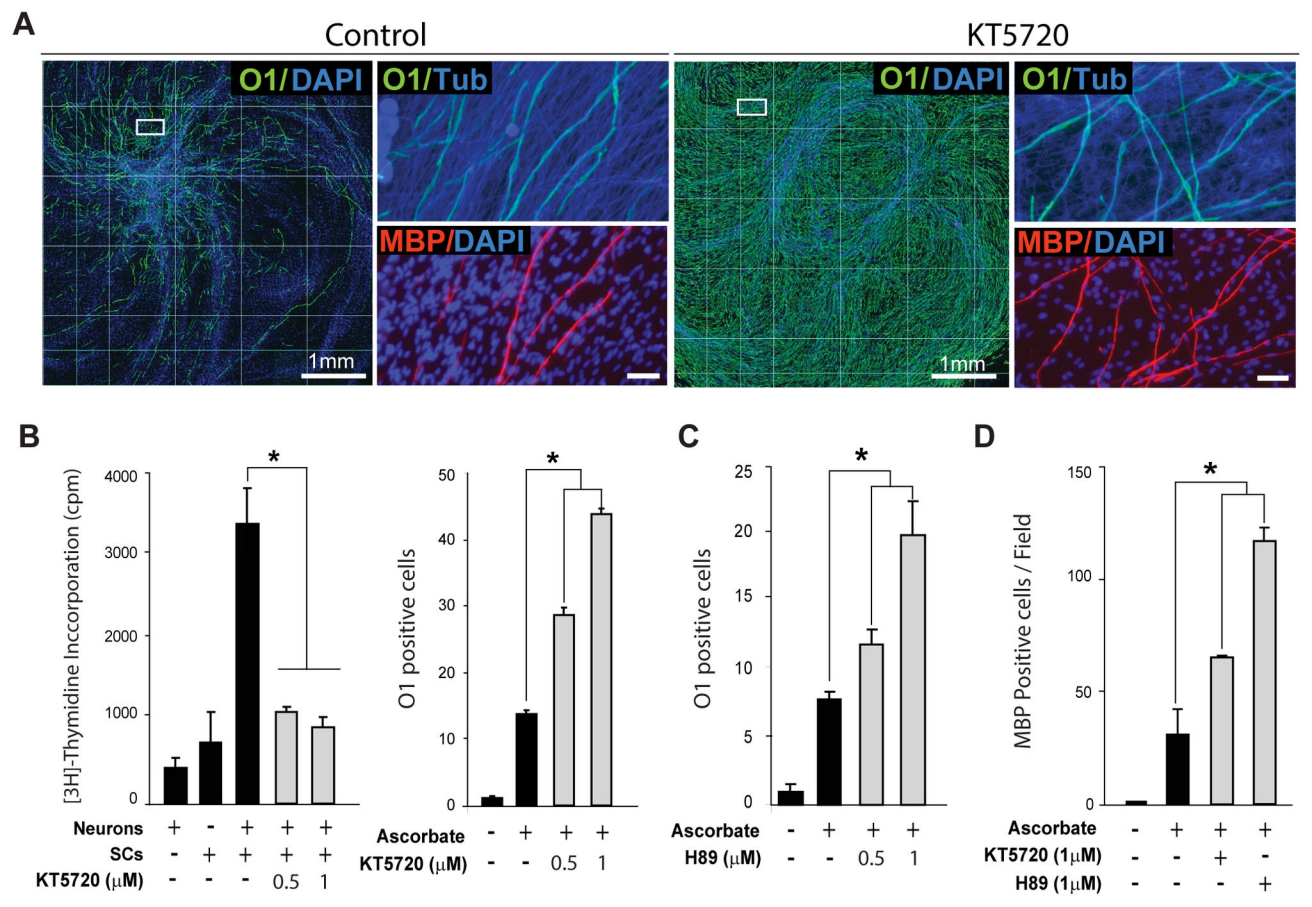
Effect of PKA inhibitors on axon contact-dependent SC proliferation and myelin sheath formation in SC-neuron cultures. SCs in co-culture with DRG neurons were induced to form myelin by addition of ascorbate-containing medium provided alone (control) or together with KT5720 or H89. Cultures were fixed 12 days after ascorbate addition (myelination phase) and analyzed by immunofluorescence microscopy (Methods). In B (left panel), SCs were seeded as a single cell suspension directly onto cultures of purified DRG neurons in the absence (control) or presence of KT5720, as indicated, and allowed to incorporate [^3^H]-thymidine for 3 days before analysis by scintillation counting. In A, myelinating SCs were identified by double immunostaining with O1 (green) and MBP (red) antibodies. MBP expression is a reliable indicator of the onset and progression of myelination in this co-culture system. The integrity of the DRG axons was confirmed by immunostaining with axon-specific βIII-tubulin antibodies (Tub, blue) and nuclei were identified with DAPI (blue). In this and all subsequent figures containing results derived from myelination assays, a composite of 10x magnification fields is shown to emphasize the relative distribution of myelinating SCs throughout the culture area (A, left panels). Ascorbate typically induces the appearance of O1 positive, MBP positive (myelinating) cells in a fraction of the SC population that is usually restricted to the area adjacent to the neuronal bodies. Selected areas (A, right panels) are shown at a higher resolution to reveal features of the myelinating cells, such as morphology and MBP expression, that cannot be appreciated at lower magnifications. Quantitative analysis of the density of O1 positive (B-C) and MBP positive cells (D) was done by automated image analysis and manual counting, respectively. In B-C, data is represented as the number of fields containing more than fifty O1 positive SCs (out of 150 microscopic fields), as we found that this measure provides a more reliable representation of the relative changes in myelination over large culture surfaces.

 Early studies showed that SCs vigorously proliferate and in turn differentiate into myelin-forming cells when stimulated by direct physical contact with axonal membranes even in the absence of an external source of cAMP [31,32]. So far, cultures consisting of SCs and primary DRG neurons are the only stable *in vitro* system capable of supporting both active proliferation and myelination by purified isolated populations of adult and postnatal rodent SCs [17,22]. Therefore, to confirm the above results in a more physiologically relevant cellular system, we used standard co-cultures of SCs and DRG neurons to compare the effect of PKA inhibitors on the proliferation and differentiation of axon-related SCs. To address the role of PKA on the ability of SCs to respond to axon contact by entering the S-phase, a single cell suspension of purified SCs was placed on top of a network of purified dissociated DRG neurons in medium containing [^3^H]-thymidine in the absence (control) or presence of PKA inhibitors. The incorporation of [^3^H]-thymidine into dividing cells was evaluated in cultures consisting of neurons and SCs alone, as controls for basal levels of tritium incorporation in each cell population, along with SCs together with neurons (Figure 4B, left panel). To address the role of PKA on myelination, SCs were allowed to proliferate extensively prior to administering differentiation medium containing ascorbate alone (control) or together with the PKA inhibitors. In the absence of ascorbate, axon-related SCs proliferate but do not differentiate or form myelin [22]. In fact, SCs maintain an immature O1 negative, MBP negative phenotype almost indefinitely in the absence of a source of ascorbate, which allows the full ensheathment of axons by SC processes along with the assembly of a basal lamina [22,33]. Myelinating cells were identified by double immuno-staining with O1 and MBP antibodies, as only those SCs that have initiated the wrapping of myelin membranes would express MBP, a late differentiation marker. Consistent with our previous observations in SC-only cultures, results indicated that KT5720 and H89 dose dependently increased the expression of O1 that occurs in response to ascorbate supplementation (Figure 4A-C) while suppressing the SC’s entrance into the S-phase induced by axonal contact (Figure 4B, shown for KT5720). Surprisingly, the synthesis of MBP positive myelin was increased in spite of prolonged exposure to the action of PKA inhibitors (Figure 4D). 

 Collectively, these results provide strong evidence that PKA activity is required for the proliferation but not the differentiation of SCs in an axonal environment conducive to myelination. 

### Selective activation of EPAC reduced SC proliferation without inducing differentiation or myelin formation

**Figure 5:**
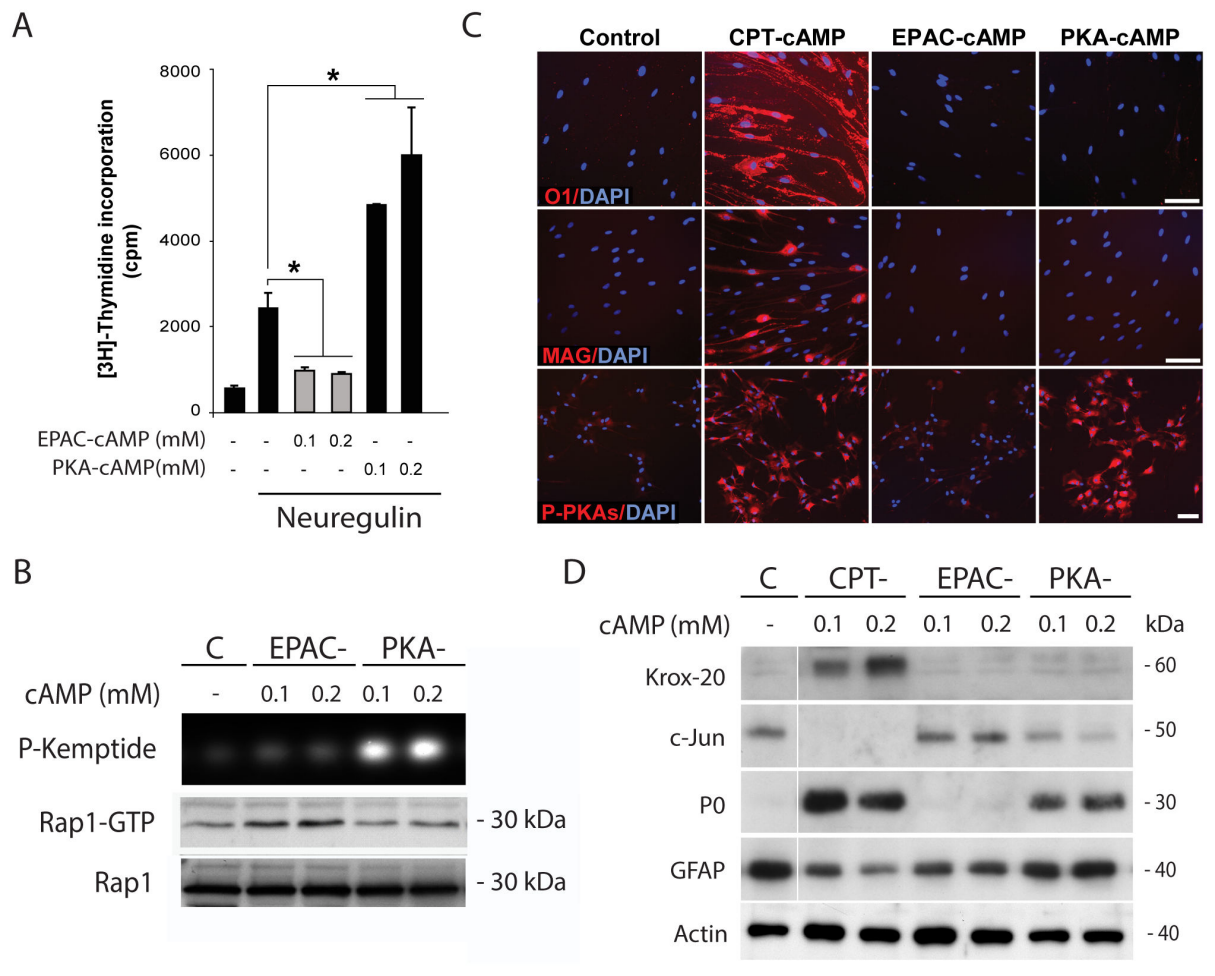
Effect of selective activation of EPAC and PKA on the proliferation and differentiation of isolated SCs. Cells were left untreated (control) or stimulated with CPT-cAMP, EPAC-cAMP or PKA-cAMP at a concentration of 200 µM each, unless otherwise indicated. Cells were analyzed for the incorporation of [^3^H]-thymidine (A) and the expression of myelin associated markers (C-D) 3 days after cAMP administration. The activity of PKA was determined by *in* *vitro* phosphorylation assays (B, upper panel) and the immunodetection of phosphorylated PKA-specific substrates (C, bottom panels) in SCs stimulated for 30 min with the indicated analogs of cAMP. The activity of EPAC (Rap1 assays) was determined under identical experimental conditions (B, lower panels). As shown in C and D, EPAC-cAMP and PKA-cAMP failed to mimic the action of CPT-cAMP (Figure 5C) or db-cAMP (Figure 2) either if provided alone or in combination (not shown). Curiously, PKA-cAMP modestly increased P_0_ and reduced c-Jun expression without increasing Krox-20 or reducing GFAP expression (D).

**Figure 6:**
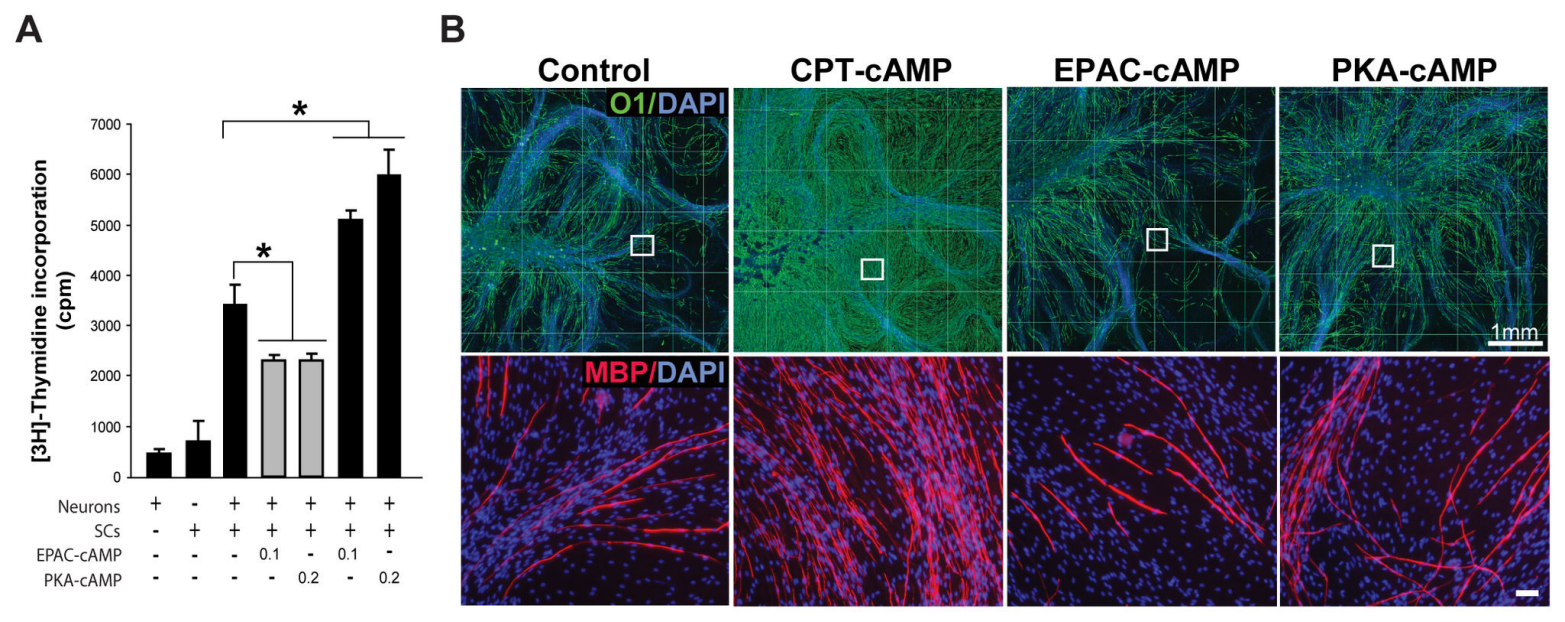
Effect of selective activation of EPAC and PKA on the proliferation and differentiation of SCs in co-culture with DRG neurons. SC-DRG neuron cultures were left untreated (control) or stimulated with CPT-cAMP, EPAC-cAMP or PKA-cAMP (200 µM each, unless otherwise indicated) for 3 days (A) or 12 days (B) prior to analysis by [^3^H]-thymidine incorporation (A) and immunofluorescence microscopy (B), respectively. Experimental conditions and analysis were identical to those of Figure 4. In B, images of myelinating co-cultures are shown at low (top panels) and high (bottom panels) magnifications, respectively. EPAC-cAMP significantly reduced proliferation of axon-contacting SCs (A). However, it did not mimic the effect of CPT-cAMP and db-cAMP (not shown) on inducing the formation of myelin sheaths (B). The strong pro-differentiating effect of CPT-cAMP is reflected by the occurrence of O1 positive, MBP positive cells throughout the co-culture system (B).

 The non-antagonistic action of PKA inhibitors on the expression of myelin-specific proteins and lipids suggested the involvement of an alternative cAMP-dependent pathway controlling the onset and progression of SC differentiation into myelinating cells. The wide variety of cAMP-dependent phenomena shown to be insensitive to PKA-selective antagonists has extensively suggested the existence of non-canonical pathways mediating the action of cAMP in mammalian cells [34]. More recently, it has become clear that some of the PKA-independent actions of cAMP are mediated by EPAC [35]. By using pathway selective analogs of cAMP, we reported opposing roles of PKA and EPAC on the proliferation of human SCs [23]. Whereas the selective activation of PKA enhances, the selective activation of EPAC decreases the mitogenic action of neuregulin. Recently, several synthetic cell permeable analogs of cAMP that potently activate EPAC but fail to activate PKA have been designed and tested as tools for the selective activation of EPAC in a variety of cell types [36]. One of the most widely used EPAC-selective cAMP derivatives is 8-(4-chlorophenylthio)-2'-O-methyladenosine-3',5'-cAMP [37]. Thus, we used this analog (herein referred to as EPAC-cAMP) to investigate the role of EPAC on the proliferation and differentiation of both isolated and axon-related SCs essentially by following a strategy similar to the one described in previous figures. The effect of EPAC-cAMP was compared to that of an equimolar dose of db-cAMP or CPT-cAMP, as these two analogs potently activated EPAC in conjunction with PKA in SCs (Figures 1B, 2A and data not shown), [23]. We also compared the effect of EPAC-cAMP with that of the PKA-selective analog N^6^-benzoyladenosine-3,5-cAMP (herein referred to as PKA-cAMP) which was used as a specificity and negative control for EPAC activation. PKA-cAMP strongly induced PKA activity in SCs, as shown by the enhancement in phospho-PKA substrate immunoreactivity (Figure 5B) and Kemptide phosphorylation *in vitro* (Figure 5C). We also confirmed the pathway-selective action of PKA-cAMP, as judged by its lack of activity towards increasing the expression of GTP-bound Rap1 in SCs (Figure 5B). EPAC-cAMP selectively activated EPAC (Figure 5B) and reduced S-phase entry in response to soluble neuregulin (Figure 5A) and axon contact stimulation (Figure 6A). Yet, EPAC-cAMP failed to mimic the differentiating action of CPT-cAMP, as evidenced by its lack of ability to: (1) increase the expression of Krox-20, O1 and MAG in SC-only cultures (Figure 5C, D); (2) reduce the expression of c-Jun and GFAP (Figure 5D); and (3) induce a morphological transformation of the cells (not shown). As opposed to CPT-cAMP, EPAC-cAMP failed to increase myelin formation in ascorbate-treated SC-neuron cultures (Figure 6B). Consistent with the action of forskolin and 6-MB-cAMP, PKA-cAMP was sufficient to synergistically enhance G1-S progression in SC-only (Figure 5A) and SC-neuron cultures (Figure 6A) without directly increasing differentiation (Figure 5C, shown for O1 and MAG expression) or myelin formation (Figure 6B, shown for O1 and MBP expression). Somehow unexpectedly, PKA-cAMP modestly increased P_0_ expression without concomitantly increasing Krox-20 or downregulating GFAP (Figure 5D). 

 Taken together, these results suggest that EPAC signaling is sufficient to reduce proliferation but not drive SC differentiation in connection to myelin formation.

### Pharmacological inhibition of EPAC antagonized cAMP-induced SC differentiation and myelin sheath formation without perturbing Krox-20 expression

**Figure 7:**
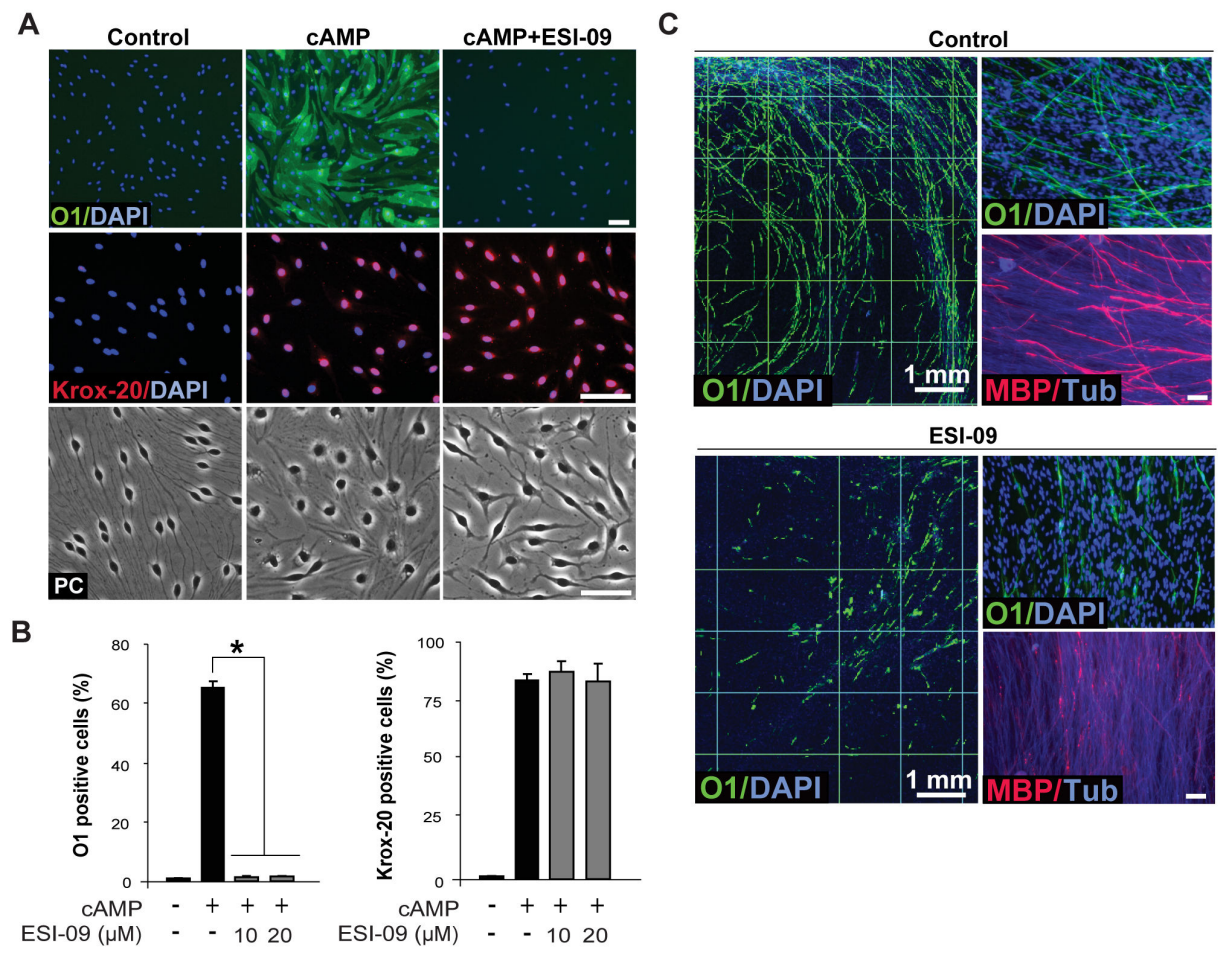
Antagonistic action of the EPAC inhibitor, ESI-09, on cAMP-induced SC differentiation and myelin sheath formation. Differentiation (A-B) and myelination (C) assays were performed and analyzed essentially as described in previous figures. Cultures were incubated with the indicated concentrations (B) or 20 µM of ESI-09 (A, C) during the whole time course of the experiments, i.e. 3 days (A-B) and 10 days (C), respectively. ESI-09 prevented the expression of O1 (A-B) and the morphological transformation that occurs in response to cAMP (CPT-cAMP, 250 µM), as revealed by the phase contrast (PC) image shown in A (bottom panel). However, ESI-09 did not prevent the expression and nuclear localization of Krox-20 (A, middle panel, and B, right panel). In C, note the dramatic reduction in the number and distribution of O1 positive and MBP positive SCs in ESI-09-treated cultures.

 Despite the availability of a number of PKA inhibitors, up until recently there were no reported EPAC antagonists to pharmacologically interfere with EPAC signaling in living cells. In the last year, novel cell permeant EPAC-specific inhibitors have become commercially available. Thus, we tested the effect of ESI-09, an inhibitor of the guanine nucleotide exchange activity of EPAC, on the induction of the expression of myelin-related markers in SCs. SCs growing in isolation and in co-culture with DRG neurons were induced to differentiate by the addition of CPT-cAMP (Figure 7A-B) and ascorbate (Figure 7C), respectively, in the absence (control) and presence of increasing concentrations of ESI-09. It has been shown that ESI-09 suppresses the cAMP-mediated activation of EPAC1 and EPAC2 but not of PKA [38]. Results shown in Figure 7 indicate that ESI-09 effectively antagonized SC differentiation induced by CPT-cAMP as well as the formation of myelin. SCs failed to express O1 and undergo the typical morphological changes induced by cAMP at all concentrations tested (Figure 7A-B). Yet, cAMP-treated SCs expressed nearly maximal levels of nuclear Krox-20 regardless of the concentration of ESI-09 used (Figure 7A-B). This observation supports the specificity of EPAC’s action on O1 expression and morphological differentiation. It also suggests that Krox-20 expression in response to cAMP relies on an EPAC-independent mechanism of action. In SC-neuron cultures, ESI-09 dramatically reduced the number of O1 positive and MBP positive SCs without compromising the health of the neurons or the SCs themselves, as revealed by staining with βIII-tubulin antibodies and DAPI, respectively (Figure 7C). 

 In summary, these results are consistent with the idea that the induction of SC differentiation and myelin sheath formation is a cAMP-dependent event that relies at least in part on EPAC activation. 

## Discussion

### Opposing roles of PKA and EPAC on the regulation of SC proliferation and differentiation

**Figure 8:**
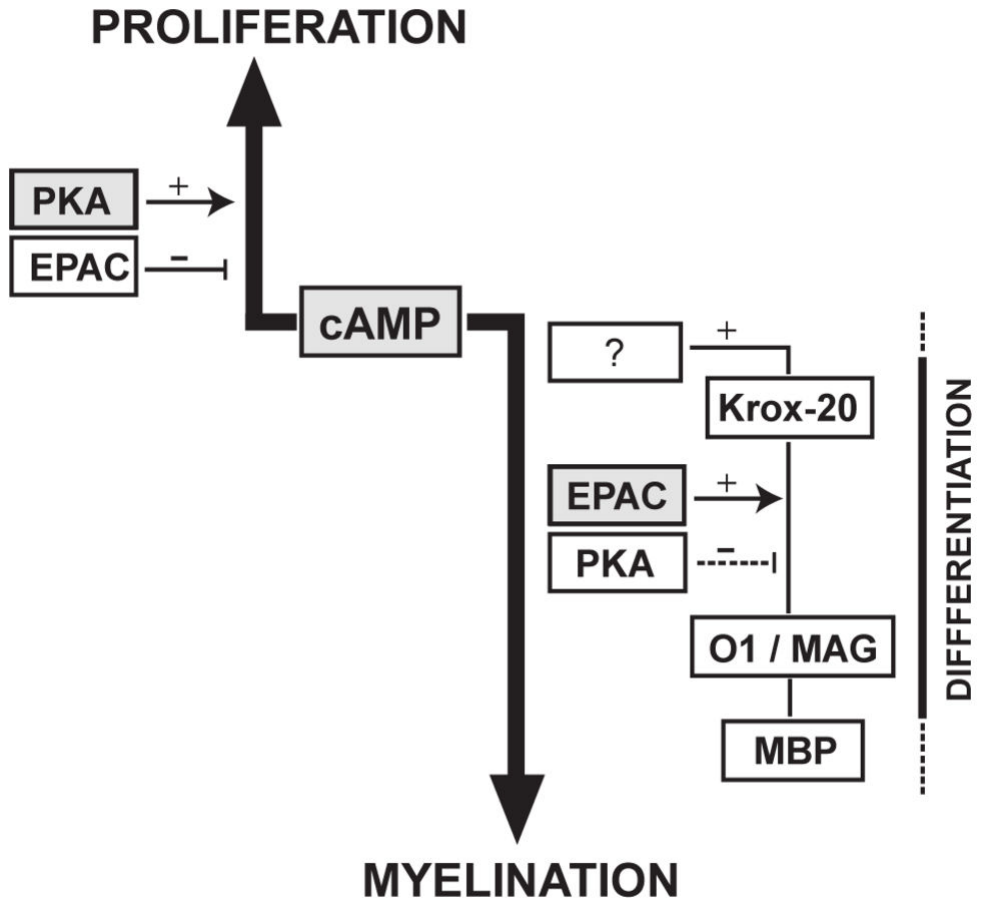
Differential contribution of PKA and EPAC to the cAMP-dependent control of proliferation and differentiation in SCs. The results derived from our *in* *vitro* studies allowed us to conclude that whereas cAMP controls proliferation through the canonical PKA pathway, differentiation into myelin-forming cells relies on an EPAC-dependent mechanism of action.

 Our studies have shown that elevated intracellular cAMP in SCs controls proliferation and differentiation via different signaling effectors. Whereas proliferation requires activation of PKA rather than EPAC, differentiation into myelin-forming cells requires activation of EPAC rather than PKA. It is therefore possible that an increase in the balance between EPAC and PKA switches the action of cAMP from a signal that enhances growth factor-induced proliferation into one that drives a program of differentiation into a myelinating phenotype. Yet, EPAC activation alone is neither sufficient to drive a full differentiating response, nor does it account for the induction of the expression of the master regulator Krox-20, altogether indicating that a still unidentified cAMP effector is likely to be required at the onset of differentiation. These novel findings have been summarized in the schematic diagram presented in Figure 8.

### PKA is a critical mediator of proliferation but not differentiation in isolated and axon-related SCs

 cAMP has been shown to control somatic cell differentiation via PKA-dependent and independent pathways depending on the type of cell and the nature of the stimulus [34]. Previous studies have suggested an involvement of PKA on the cAMP-induced differentiation of isolated SCs [39], the expression of the myelinating SC marker E-cadherin [40] and the formation of myelin in SC-neuron cultures [13,41]. Even though we cannot rule out a role of PKA at some stage in the differentiation process, our collective data provided no clear indication that PKA is part of the signal transduction mechanism used by cAMP to change the balance Krox-20/c-Jun, induce the expression of critical myelin-related markers or form MBP positive myelin *in vitro*. On one hand, results using PKA-selective analogs of cAMP showed no correlation between the magnitude of the PKA activity signal and the induction of differentiation. On the other hand, it was interesting to observe an increased rather than decreased expression of Krox-20, O1, MAG, P_0_ and even MBP under conditions in which PKA activity was pharmacologically suppressed. This observation leads to the interesting but yet to be explored possibility that PKA exerts a negative regulation on the myelination process. The PKA-independent control of SC differentiation was confirmed by using a battery of PKA agonists and antagonists acting through different molecular mechanisms. Results were also validated by using isolated adult and postnatal SCs in combination with organotypic SC-neuron cultures that faithfully recapitulate essential features of SC development including axon contact-stimulated proliferation and the formation of myelin sheaths [22]. In addition, proliferation and differentiation assays were run in parallel cultures to confirm the bioactivity of the drugs used. 

 Our results are in agreement with previous findings indicating that PKA is required and sufficient to mediate the effect of cAMP on SC mitogenesis [23,27]. The non-antagonistic action of PKA inhibitors on MBP expression brings strong support to the concept that the differentiation of SCs into a myelinating phenotype relies on a non-canonical cAMP-dependent mechanism of action that is independent of the one that controls proliferation. 

### EPAC is a critical mediator of differentiation and myelin formation in SCs

 Our collective data have shown that an alternative cAMP-dependent pathway mediated by EPAC is responsible at least in part for the pro-myelinating action of cAMP in SCs. We arrived to this conclusion after studying the effects of EPAC-selective activators and inhibitors on the differentiation of isolated and axon-related SCs. First, studies using forskolin, which activates PKA but not EPAC in SCs, as well as PKA-selective analogs of cAMP, revealed that SCs do not progress into a fully differentiated state in the absence of EPAC activation. Second, inhibition of EPAC activity prevented not only the morphological differentiation that occurs in response to cAMP but also the expression of O1 and MBP. Even though EPAC seems to be a pivotal mediator of differentiation required for the expression of proteins and lipids specific to the myelin sheath, our data indicates that it still not sufficient to drive all aspects of differentiation. In fact, we found that EPAC is neither sufficient nor required for the cAMP-dependent expression of Krox-20, one of the earliest traceable events during cAMP-dependent differentiation. An open question is how cAMP differentially controls PKA and EPAC activation during proliferation and differentiation. cAMP is produced locally within discrete intracellular microdomains that may differ according to the nature of the stimulus and the cellular state [42]. Consequently, it is possible that the proximity of a given effector to a cellular compartment with locally increased cAMP may determine whether PKA or EPAC is preferentially activated. 

 Other examples in the literature have shown that not all PKA-independent events rely exclusively on EPAC signaling. Indeed, it has also become apparent that alternative pathways to the ones mediated by PKA and EPAC should exist to transduce cAMP signals in mammalian cells. In the neuronal cell line PC12, the cAMP-dependent control of neurite outgrowth by pituitary AC-activating peptide (PACAP) illustrates a similar type of non-canonical regulation by cAMP signals, as the induction of neuritogenesis is both insensitive to PKA antagonists and only partially transduced by EPAC [43]. The overall control of proliferation is also similar in SCs and PC12 cells, as they both respond to PKA- and EPAC-selective agonists by increasing and decreasing their proliferation rate, respectively [44]. 

 How EPAC controls differentiation is not known as we have found no clear evidence that EPAC activity directly induces or modulates the activation of ErbB, MEK-ERK or Akt in SCs [23]. Another open question is how Krox-20 expression is increased in response to signals elevating cAMP. Despite the fact that several cAMP-responsive genes were shown to be regulated in a PKA-independent manner, very little is known about their activation by specific transcription factors [34]. Even less is known about how these transcription factors are regulated via PKA-independent cAMP sensing. This highlights the importance of the identification of Krox-20 as a novel target for regulation by non-canonical cAMP signals. Of note, another Krox/Egr family member, Egr1, has been shown to mediate cAMP-induced neuritogenesis independently of PKA [45]. 

 Some unexpected results have indeed emphasized the complexity underlying the control of myelin gene expression in response to cAMP. One example is the expression of P_0_, a major structural component of peripheral myelin, which we found to be sensitive to the levels of cAMP but not Krox-20. In fact, evidence suggests that Krox-20 may mediate only partially the effect of cAMP on differentiation. The regulation of periaxin expression in SCs provides an additional example of another myelin-related protein whose expression depends on cAMP rather than Krox-20 [46].

### Integration of cAMP and neuregulin-ErbB signals in the control of SC proliferation and differentiation

 It is well-recognized that cAMP uses PKA and EPAC to either directly transduce signals in the cytoplasm and nucleus or to modulate the intensity and duration of signaling occurring through other pathways, a mechanism of regulation defined as “gating by cAMP” [47]. We previously performed a comprehensive analysis of the molecular mechanism that underlies the synergistic regulation of SC proliferation by cAMP and showed that PKA synergizes S-phase entry because it enhances the ligand-dependent activation of the neuregulin co-receptor ErbB2-ErbB3 [23] and downstream signaling through MEK-ERK and Akt pathways [7]. This type of signaling cross-talk between neuregulin-ErbB and cAMP-PKA signaling is a clear example of cAMP-dependent gating. In SCs, the gating effect is mediated by the direct phosphorylation of ErbB2 by PKA on a highly conserved regulatory threonine residue, Thr-686. The phosphorylation of this site is in turn required for the ability of cAMP to synergistically enhance the tyrosine phosphorylation of ErbB2 and ErbB3, the activation of ERK and Akt and the induction of proliferation in response to neuregulin [23]. On the contrary, evidence indicates that cAMP can effectively drive myelin gene expression even in the absence of other signals [19], which suggests that the effect of cAMP on SC differentiation is most likely direct. Indeed, we have found that cAMP elevation is sufficient to not only initiate but also maintain a state of differentiation, as SCs rapidly revert to an undifferentiated state upon the simple removal of the cAMP stimulus either in the absence or presence of mitogenic factors [20]. 

 A long-standing concept has been that both SC proliferation and myelination occur in response to signals derived from axons. The expression of neuregulin isoforms on the axonal surface mediates activation of ErbB receptors in SCs and axon contact-dependent proliferation and migration [48,49]. In addition, an extensive body of data has shown that the levels of axon-bound neuregulin-1 type III are key determinants for regulating myelin thickness in peripheral nerves (reviewed in 50). Even though the extracellular signals and membrane receptors that induce cAMP elevation in SCs have not yet been identified, different lines of evidence support the idea that neuregulin-ErbB and cAMP exert their effects on differentiation through different and independent mechanisms. Similar to other RTK ligands, neuregulin is a fairly poor activator of AC-cAMP in mammalian cells in general. The recent discovery of Gpr126, an orphan G protein-coupled receptor that is required for myelination, may provide additional clues on how this process is controlled by signals activating AC-cAMP. Deletion of Gpr126 arrests SCs at an early pre-myelinating Krox-20 negative stage in both zebrafish and mammals [51,52]. Because the effect of the Gpr126 deletion is rescued by administration of forskolin, it is possible that this orphan receptor mediates its effects on peripheral myelination through activation of AC and cAMP. Besides Gpr126, other components of the cAMP signaling pathway that have been reported to control myelination are the myosin light chain kinase [53] and Rac1 [54].

 To conclude, the results presented here have indicated that SCs differentiate in response to AC-cAMP signals that are transduced non-canonically though an EPAC-dependent mechanism. As shown for PC12 cells, a great challenge still remains to identify novel intracellular transducers for non-canonical cAMP pathways controlling cell differentiation in general. cAMP seems to fulfill all basic requirements for a candidate intracellular signal that initiates differentiation towards a myelinating phenotype. The identification of the source and chemical nature of the ligand for Gpr126 will unveil the functional connection of this GPCR to the cAMP system. It will also help understand whether Gpr126 mediates its effects through EPAC and/or other cAMP-dependent effector pathway. Yet, a better understanding of how cAMP signals are transduced and integrated with other signals such as those elicited by neuregulin isoforms and their receptors will provide clues on the mechanistic control underlying the timing and progression of differentiation in connection to myelin sheath formation in peripheral nerves. 

## Materials and Methods

### Ethics statement

 All procedures using animals were approved by the University of Miami Animal Care and Use Committee.

### Materials

 N^6^ 2'- O- dibutyryladenosine- 3', 5'- cyclic monophosphate (db-cAMP), 8-(4-Chlorophenylthio) adenosine-3',5'-cyclic monophosphate (CPT-cAMP), N^6^-monobutyryladenosine-3',5'-cyclic monophosphate (6MB-cAMP), 8-(4-chlorophenylthio)-2'-O-methyladenosine-3',5'-cyclic monophosphate (EPAC-cAMP), N^6^-benzoyladenosine-3,5-cyclic monophosphate (PKA-cAMP), adenosine-3',5'-cyclic monophosphorothioate, *Rp*-isomer (*Rp*-cAMP), 8-(4-Chlorophenylthio) guanosine-3', 5'-cyclic monophosphate (CPT-cGMP) and the non-cyclic nucleotide EPAC antagonist (ESI-09) were from Biolog (US distributor, Axxora LLC, San Diego, CA). Recombinant heregulin-β1177-244 (hereafter referred to as “neuregulin”) was from Genentech (South San Francisco, CA). Forskolin and β-actin antibodies were from Sigma (St. Louis, MO). GFAP antibodies were from DAKO (Carpinteria, CA). Myelin-associated glycoprotein (MAG), protein zero (P_0_), myelin basic protein (MBP), and βIII-tubulin antibodies were from Chemicon (Temecula, CA). c-Jun antibodies were from Santa Cruz Biotech (Santa Cruz, CA). *Myr*-PKI was from Biomol (Plymouth Meeting, PA). H89 was from Calbiochem-Novabiochem Corp. (La Jolla, CA) and KT5720 from Enzo Life Sciences. [^3^H]-thymidine (6.7 Ci/mmol) and SolvableTM were from Perkin-Elmer (Boston, MA). O1 hybridoma cells [16] were kindly provided by Dr. Melitta Schachner (Rutgers, Piscataway, NJ) and Krox-20 antibodies by Dr. Dies Meijer (Erasmus University Medical Center, Rotterdam).

### Primary cultures of adult and postnatal SCs

 Rat SCs were prepared from adult sciatic nerves by a modification of a previously reported method [17]. Briefly, the sciatic nerve tissue was cut into small segments and allowed to degenerate *in vitro* by incubation in DMEM medium containing 10% heat inactivated fetal bovine serum (FBS) for 10 days. Degenerated nerve explants were dissociated with a mixture of 0.25% dispase and 0.05% collagenase and the resulting cell suspension was plated on poly-L-lysine (PLL)-coated dishes in DMEM-10% FBS. Contaminating fibroblasts were removed by a complement reaction using Thy 1.1 antibodies (ATCC, Manassas, VA). The purified SCs were expanded in DMEM-10% FBS medium supplemented with 2 µM forskolin, 20 µg/ml bovine pituitary extract (Biomedical Tech., Stoughton, MA), and 10 nM neuregulin. SCs obtained by this method are fully competent to myelinate axons from DRG neurons *in vitro* [17].

 SCs from postnatal day one rat sciatic nerves were established essentially as described previously [18]. The nerve tissue was dissociated by sequential incubation with 0.1% collagenase and 0.25% trypsin and the resulting cell suspension was purified of contaminating fibroblasts by growth in DMEM-10% FBS containing 10 µM cytosine arabinoside for 3 days. Postnatal SCs were expanded in number essentially as described for adult SCs. Experimental conditions were tested using early passage SCs (2 to 4 rounds of expansion) plated on 24-well dishes coated with PLL-laminin. SC cultures (adult and postnatal) consisted of >98% SCs based on immunostaining with the SC marker S100. For practical purposes (i.e. cell yields / preparation), most experiments were performed using cultures of adult SCs. Postnatal SCs were used as an alternative model system for result validation. Of note, we have previously compared the adult and postnatal (rat) SC populations and found no relevant differences in their responses to cAMP or growth factors [19,20].

### Primary cultures of dissociated DRG neurons

 Cultures of purified embryonic dissociated DRG neurons were established following standard methods [21]. The DRG bodies were dissected from rat embryos on the 15^th^ day of gestation and then dissociated with 0.25% trypsin (37°C, 45 min) followed by gentle trituration. The resulting cell suspensions (50 μl drops containing 5,000-20,000 cells) were plated in the center of a well from a laminin-coated 24-well dish (BD Biosciences). Cultures were purified of non-neuronal cells by treatment with the anti-mitotic agent 5-fluoro-2'deoxyuridine (10 µM), which was added to the cell culture medium one day after cell plating. The neuronal cultures were established and maintained in Neurobasal medium containing B27 supplement (Invitrogen, Carlsbad, CA), 10 ng/ml nerve growth factor (R&D Systems) and 1 mM L-glutamine (Life Technologies). DRG neurons were used for experimentation 7-10 days after plating, which is the usual time required for establishing pure neuronal cultures with radiating axons all over the available surface.

### Proliferation assays for isolated and axon-related SCs

 The incorporation of [^3^H]-thymidine into nuclear DNA was assayed as a measure of S-phase entry, as reported previously [7]. Briefly, SCs were plated at a density of 100,000 cells/well on PLL-laminin-coated 24-well dishes. To reduce the rate of proliferation to basal levels, the cells were deprived of mitogens and serum by sequential incubation in DMEM-10% FBS (1 day) and DMEM-1% FBS (1 day) prior to mitogenic treatment and addition of [^3^H]-thymidine (0.25 µCi/well). In some experiments, a mitogenic concentration of forskolin (2 µM) was used to enhance growth factor-induced SC proliferation, and thereby achieve a maximal response in [^3^H]-thymidine incorporation [7]. 

 Axon contact-induced SC proliferation was measured by plating purified SCs on purified dissociated DRG neurons in the presence of [^3^H]-thymidine. For co-culture initiation, subconfluent cultures of mitogen- and serum-starved SCs were trypsin-dissociated to obtain a single cell suspension (usually 100,000 cells) prior to being placed on top of cultures of purified DRG neurons [21]. To prevent possible detrimental effects of the kinase inhibitors on the initial interaction between SCs and axons, SCs were allowed to attach to and extend their processes along the axonal surface for 4 hours prior to the addition of inhibitors and [^3^H]-thymidine. The incorporation of tritium into the proliferating SCs was determined by liquid scintillation counting 2-3 days after mitogenic stimulation or co-culture initiation. Stocks of cAMP analogs were prepared in DMEM and inhibitors in DMSO, according to the manufacturer’s instructions. The final concentration of DMSO in the culture medium was calculated to be less than 0.2%. Cells in the control condition were stimulated with the appropriate volume of vehicle (i.e. DMEM or DMSO). 

### Differentiation assays for isolated SCs

 SCs were differentiated by treatment with phosphodiesterase (PDE)-resistant cell permeable analogs of cAMP. Throughout these studies, we indistinctively used the cAMP derivatives CPT-cAMP and db-cAMP provided in the range of 0.1-0.3 mM (isolated SCs) or 10-20 μM (SC-neuron cultures) on the basis of their demonstrated effectiveness to increase the expression of myelination-associated markers [19]. Cultures were analyzed by western blot or immunofluorescence microscopy 3-4 days after treatment, unless otherwise indicated. SC cultures maintained from the outset in the absence of cAMP-inducing agents served as negative controls for the expression of myelination-associated markers. To simplify the interpretation of results, proliferation and differentiation assays were typically carried out simultaneously in sibling cultures maintained in basal medium (DMEM-1% FBS) for at least 1 day prior to stimulation. Of note, cultured SCs not exposed to cAMP typically display an immature proliferative phenotype which is denoted by negligible levels of expression of myelin-associated proteins and lipids. Throughout these studies, we have concluded that SCs have differentiated into a myelinating SC-related phenotype if there was an observable: 1) increase in the expression of myelinating SC markers; 2) decrease in the expression of immature SC markers; 3) cell shape transformation that includes cell enlargement and acquisition of an epithelial-like morphology, and 4) post-mitotic state, as assessed by an inability of the SCs to respond to growth factors by re-entering the cell cycle.

### Myelin formation assays

 To generate myelinating co-cultures of adult SCs and DRG neurons, a single cell suspension of SCs was seeded on top of a network of pure DRG neurons and allowed to repopulate the axons (proliferation phase) before inducing myelination by the addition of ascorbate (50 µg/ml) and 5% FBS (myelination phase) [22]. Cell culture studies have shown that SCs require repeated cycles of cell division prior to becoming competent to myelinate axons [21]. Medium containing ascorbate was changed on a 3-day basis up until fixation and immunostaining for the myelin markers O1 and MBP. SC-neuron cultures were also routinely co-stained with antibodies against βIII-tubulin (neuronal marker) and DAPI (nuclear marker), which served as reference controls for the extension of the axonal web and the location of the cells, respectively. Myelinating SCs were identified by their characteristic O1 positive, MBP positive phenotype. SC-neuron cultures maintained in medium without ascorbate served as controls for non-myelinating co-cultures.

 Quantification of myelinating SCs was performed by automated fluorescence microscopy using a Thermo Scientific Cellomics ArrayScan VTI High Content System Reader, version 6.6.2.0 (High Content Screening Core Facility, The Miami Project to Cure Paralysis). Low magnification images (10x) of O1 (488) and DAPI (UV) staining were taken as serial images starting from the center toward the periphery of the well (24-well format). The number of O1 positive cells/condition was calculated in reference to the total number of cells (DAPI) using Target Activation Bioapplication software. An average of 150 microscopic fields (about one fourth of the well’s surface) was routinely scanned for analysis. The number of MBP positive myelin segments was done by manual counting. All experimental conditions were analyzed in triplicate samples. 

### Immunofluorescence microscopy

 Cultures were fixed by sequential treatment with 4% paraformaldehyde and –20°C methanol. Cultures were blocked in 5% normal goat serum in PBS, incubated overnight at 4°C with the appropriate dilution of the primary antibody and rinsed three times with PBS prior to incubation with Alexa-conjugated secondary antibodies. Cell surface labeling with O1 antibodies was done by incubating live cultures with hybridoma culture supernatant (20 min, RT) before fixation. Cells were mounted with Vectashield containing DAPI (Vector Labs, Burlingame, CA) and analyzed by conventional fluorescence microscopy. Images were taken using a cooled digital CCD camera (SensiCam QE, Cooke Corp.) coupled to an Olympus IX70 inverted fluorescence microscope. Black and white images (8 bit, tiff format) were artificially colorized in RGB format, digitally processed and arranged for presentation using Adobe Photoshop V7.0 and Adobe Illustrator CS3. For cell quantification analysis, pictures from random fields were taken at low magnification (10x) and the number of cells labeled positive for the indicated markers was determined in reference to the total number of cells (DAPI staining). Cells were classified as positive or negative for the expression of Krox-20 (nuclear localization), O1/MAG (membrane localization) and MBP (myelin segments) in comparison to non-treated controls and without regard to the variability of staining intensity shown by individual cells. At least 1,000 cells were analyzed in each sample.

### PKA and EPAC activity assays

 To determine the relative changes in the levels of PKA activity in individual SCs, we assessed the phosphorylation of specific PKA substrates by immunoflouorescence microscopy. For this, methanol-fixed cells were incubated (4 h, RT) with antibodies recognizing PKA-specific phospho-motifs ([RR]-x-[S*/T*]) in target proteins (Cell Signaling Tech, Beverly, MA). It has been shown previously that this antibody detects both cytoplasmic and nuclear phosphorylated PKA substrates in SCs and that the extent of phospho-PKA substrate immunoreactivity is directly proportional to the levels of PKA activation and intracellular cAMP [23]. The activity of PKA was also determined in samples of lysed cells by *in vitro* phosphorylation of the Kemptide substrate, a PKA-specific fluorescent peptide of sequence LRRASLG (Promega). 

 To measure the nucleotide exchange activity of EPAC, we assayed the GTP-binding activity of its downstream effector, Rap1. For this, we used a standard method that involves the precipitation of GTP-bound Rap1 molecules by affinity binding to purified recombinant Ral-GDS-agarose beads, according to the manufacturer’s recommendations (Roche). Purified samples containing GTP-bound Rap1 were denatured in SDS loading buffer, resolved by 15% denaturing polyacrylamide gel electrophoresis (SDS-PAGE) and analyzed by western blotting using anti-Rap1 antibodies (Santa Cruz). Samples from total cell lysates were also analyzed as controls for total Rap1 expression. 

### Western blots

 Total cell lysates were prepared as reported previously [20]. Equal protein samples (typically 5 µg protein/lane) were subjected to 8-10% SDS-PAGE and then transferred to polyvinylidene fluoride membranes (Millipore, Bedford, MA). Membranes were blocked with ECL blocking agent (GE Healthcare) in Tris-Buffer saline containing 0.05% Tween-20 (TBS-T) and incubated overnight with a 1:1000 dilution of each primary antibody. The membranes were washed 3 times with TBS-T prior to incubation with horseradish peroxidase (HRP)-conjugated secondary antibodies (Promega). Immunoreactive protein bands were detected by enhanced chemiluminescence (ECL) using ECL Advanced or ECL Plus (GE Healthcare) depending on signal intensity. The expression of β-actin was used as a loading and reference control. 

### Statistical analysis

 The statistical significance of the different treatments was analyzed relative to each control group by a one-way analysis of variance (ANOVA) followed by a post-hoc Tukey test using IBM SPSS statistics software. A p-value <0.05 was interpreted as statistically significant. Data from a representative experiment out of at least three independent experiments performed are shown. 

## References

[1] Jessen KR, Mirsky R, Morgan L (1991) Role of cyclic AMP and proliferation controls in Schwann cell differentiation. Ann N Y Acad Sci 633: 78-89. doi:10.1111/j.1749-6632.1991.tb15597.x. PubMed: 1665043. 1665043

[2] Morgan L, Jessen KR, Mirsky R (1991) The effects of cAMP on differentiation of cultured Schwann cells: progression from an early phenotype (04+) to a myelin phenotype (P0+, GFAP-, N-CAM-, NGF-receptor-) depends on growth inhibition. J Cell Biol 112: 457-467. doi:10.1083/jcb.112.3.457. PubMed: 1704008. 1704008 PMC2288828

[3] Sobue G, Pleasure D (1984) Schwann cell galactocerebroside induced by derivatives of adenosine 3',5'-monophosphate. Science 224: 72-74. doi:10.1126/science.6322307. PubMed: 6322307. 6322307

[4] Raff MC, Hornby-Smith A, Brockes JP (1978) Cyclic AMP as a mitogenic signal for cultured rat Schwann cells. Nature 273: 672-673. doi:10.1038/273672a0. PubMed: 207999. 207999

[5] Rahmatullah M, Schroering A, Rothblum K, Stahl RC, Urban B et al. (1998) Synergistic regulation of Schwann cell proliferation by heregulin and forskolin. Mol Cell Biol 18: 6245-6252. PubMed: 9774641. 9774641 10.1128/mcb.18.11.6245 PMC109211

[6] Kim HA, Ratner N, Roberts TM, Stiles CD (2001) Schwann cell proliferative responses to cAMP and Nf1 are mediated by cyclin D1. J Neurosci 21: 1110-1116. PubMed: 11160381. 11160381 10.1523/JNEUROSCI.21-04-01110.2001 PMC6762237

[7] Monje PV, Bartlett Bunge M, Wood PM (2006) Cyclic AMP synergistically enhances neuregulin-dependent ERK and Akt activation and cell cycle progression in Schwann cells. Glia 53: 649-659. doi:10.1002/glia.20330. PubMed: 16470843. 16470843

[8] Porter S, Clark MB, Glaser L, Bunge RP (1986) Schwann cells stimulated to proliferate in the absence of neurons retain full functional capability. J Neurosci 6: 3070-3078. PubMed: 3760949. 3760949 10.1523/JNEUROSCI.06-10-03070.1986 PMC6568804

[9] Topilko P, Schneider-Maunoury S, Levi G, Baron-Van Evercooren A, Chennoufi AB et al. (1994) Krox-20 controls myelination in the peripheral nervous system. Nature 371: 796-799. doi:10.1038/371796a0. PubMed: 7935840. 7935840

[10] Monuki ES, Weinmaster G, Kuhn R, Lemke G (1989) SCIP: a glial POU domain gene regulated by cyclic AMP. Neuron 3: 783-793. doi:10.1016/0896-6273(89)90247-X. PubMed: 2561978. 2561978

[11] Lemke G, Kuhn R, Monuki ES, Weinmaster G (1991) Expression and activity of the transcription factor SCIP during glial differentiation and myelination. Ann N Y Acad Sci 633: 189-195. doi:10.1111/j.1749-6632.1991.tb15609.x. PubMed: 1665030. 1665030

[12] Zorick TS, Syroid DE, Arroyo E, Scherer SS, Lemke G (1996) The Transcription Factors SCIP and Krox-20 Mark Distinct Stages and Cell Fates in Schwann. Cell Differentiation - Mol Cell Neurosci 8: 129-145. 10.1006/mcne.1996.0052 8954628

[13] Yoon C, Korade Z, Carter BD (2008) Protein kinase A-induced phosphorylation of the p65 subunit of nuclear factor-kappaB promotes Schwann cell differentiation into a myelinating phenotype. J Neurosci 28: 3738-3746. doi:10.1523/JNEUROSCI.4439-07.2008. PubMed: 18385332. 18385332 PMC6671072

[14] Parkinson DB, Bhaskaran A, Arthur-Farraj P, Noon LA, Woodhoo A et al. (2008) c-Jun is a negative regulator of myelination. J Cell Biol 181: 625-637. doi:10.1083/jcb.200803013. PubMed: 18490512. 18490512 PMC2386103

[15] de Rooij J, Zwartkruis FJ, Verheijen MH, Cool RH, Nijman SM et al. (1998) Epac is a Rap1 guanine-nucleotide-exchange factor directly activated by cyclic AMP. Nature 396: 474-477. doi:10.1038/24884. PubMed: 9853756. 9853756

[16] Sommer I, Schachner M (1981) Monoclonal antibodies (O1 to O4) to oligodendrocyte cell surfaces: an immunocytological study in the central nervous system. Dev Biol 83: 311-327. doi:10.1016/0012-1606(81)90477-2. PubMed: 6786942. 6786942

[17] Morrissey TK, Kleitman N, Bunge RP (1991) Isolation and functional characterization of Schwann cells derived from adult peripheral nerve. J Neurosci 11: 2433-2442. PubMed: 1869923. 1869923 10.1523/JNEUROSCI.11-08-02433.1991 PMC6575499

[18] Brockes JP, Fields KL, Raff MC (1979) Studies on cultured rat Schwann cells. I. Establishment of purified populations from cultures of peripheral nerve. Brain Res 165: 105-118. doi:10.1016/0006-8993(79)90048-9. PubMed: 371755. 371755

[19] Monje PV, Rendon S, Athauda G, Bates M, Wood PM et al. (2009) Non-antagonistic relationship between mitogenic factors and cAMP in adult Schwann cell re-differentiation. Glia 57: 947-961. doi:10.1002/glia.20819. PubMed: 19053056. 19053056 PMC2829776

[20] Monje PV, Soto J, Bacallao K, Wood PM (2010) Schwann cell dedifferentiation is independent of mitogenic signaling and uncoupled to proliferation: role of cAMP and JNK in the maintenance of the differentiated state. J Biol Chem 285: 31024-31036. doi:10.1074/jbc.M110.116970. PubMed: 20634285. 20634285 PMC2945593

[21] Wood PM (1976) Separation of functional Schwann cells and neurons from normal peripheral nerve tissue. Brain Res 115: 361-375. doi:10.1016/0006-8993(76)90355-3. PubMed: 135599. 135599

[22] Eldridge CF, Bunge MB, Bunge RP, Wood PM (1987) Differentiation of axon-related Schwann cells in vitro. I. Ascorbic acid regulates basal lamina assembly and myelin formation. J Cell Biol 105: 1023-1034. doi:10.1083/jcb.105.2.1023. PubMed: 3624305. 3624305 PMC2114758

[23] Monje PV, Athauda G, Wood PM (2008) Protein kinase A-mediated gating of neuregulin-dependent ErbB2-ErbB3 activation underlies the synergistic action of cAMP on Schwann cell proliferation. J Biol Chem 283: 34087-34100. doi:10.1074/jbc.M802318200. PubMed: 18799465. 18799465 PMC2590688

[24] Yamada H, Komiyama A, Suzuki K (1995) Schwann cell responses to forskolin and cyclic AMP analogues: comparative study of mouse and rat Schwann cells. Brain Res 681: 97-104. doi:10.1016/0006-8993(95)00293-Y. PubMed: 7552298. 7552298

[25] Kopperud R, Krakstad C, Selheim F, Døskeland SO (2003) cAMP effector mechanisms. Novel twists for an 'old' signaling system. FEBS Lett 546: 121-126. doi:10.1016/S0014-5793(03)00563-5. PubMed: 12829247. 12829247

[26] Sobue G, Shuman S, Pleasure D (1986) Schwann cell responses to cyclic AMP: proliferation, change in shape, and appearance of surface galactocerebroside. Brain Res 362: 23-32. doi:10.1016/0006-8993(86)91394-6. PubMed: 3002553. 3002553

[27] Kim HA, DeClue JE, Ratner N (1997) cAMP-dependent protein kinase A is required for Schwann cell growth: interactions between the cAMP and neuregulin/tyrosine kinase pathways. J Neurosci Res 49: 236-247. doi:10.1002/(SICI)1097-4547(19970715)49:2. PubMed: 9272646. 9272646

[28] Gadbois DM, Crissman HA, Tobey RA, Bradbury EM (1992) Multiple kinase arrest points in the G1 phase of nontransformed mammalian cells are absent in transformed cells. Proc Natl Acad Sci U S A 89: 8626-8630. doi:10.1073/pnas.89.18.8626. PubMed: 1528872. 1528872 PMC49973

[29] Lochner A, Moolman JA (2006) The many faces of H89: a review. Cardiovasc Drug Rev 24: 261-274. doi:10.1111/j.1527-3466.2006.00261.x. PubMed: 17214602. 17214602

[30] Glass DB, Cheng HC, Mende-Mueller L, Reed J, Walsh DA (1989) Primary structural determinants essential for potent inhibition of cAMP-dependent protein kinase by inhibitory peptides corresponding to the active portion of the heat-stable inhibitor protein. J Biol Chem 264: 8802-8810. PubMed: 2722799. 2722799

[31] Wood PM, Bunge RP (1975) Evidence that sensory axons are mitogenic for Schwann cells. Nature 256: 662-664. doi:10.1038/256662a0. PubMed: 1171378. 1171378

[32] Salzer JL, Bunge RP, Glaser L (1980) Studies of Schwann cell proliferation. III. Evidence for the surface localization of the neurite mitogen. J Cell Biol 84: 767-778. doi:10.1083/jcb.84.3.767. PubMed: 6153659. 6153659 PMC2110586

[33] Eldridge CF, Bunge MB, Bunge RP (1989) Differentiation of axon-related Schwann cells in vitro: II. Control of myelin formation by basal lamina. J Neurosci 9: 625-638. PubMed: 2918381. 2918381 10.1523/JNEUROSCI.09-02-00625.1989 PMC6569783

[34] Sands WA, Palmer TM (2008) Regulating gene transcription in response to cyclic AMP elevation. Cell Signal 20: 460-466. doi:10.1016/j.cellsig.2007.10.005. PubMed: 17993258. 17993258

[35] Gloerich M, Bos JL (2010) Epac: defining a new mechanism for cAMP action. Annu Rev Pharmacol Toxicol 50: 355-375. doi:10.1146/annurev.pharmtox.010909.105714. PubMed: 20055708. 20055708

[36] Holz GG, Chepurny OG, Schwede F (2008) Epac-selective cAMP analogs: new tools with which to evaluate the signal transduction properties of cAMP-regulated guanine nucleotide exchange factors. Cell Signal 20: 10-20. doi:10.1016/j.cellsig.2007.07.009. PubMed: 17716863. 17716863 PMC2215344

[37] Enserink JM, Christensen AE, de Rooij J, van Triest M, Schwede F et al. (2002) A novel Epac-specific cAMP analogue demonstrates independent regulation of Rap1 and ERK. Nat Cell Biol 4: 901-906. doi:10.1038/ncb874. PubMed: 12402047. 12402047

[38] Almahariq M, Tsalkova T, Mei FC, Chen H, Zhou J et al. (2013) A novel EPAC-specific inhibitor suppresses pancreatic cancer cell migration and invasion. Mol Pharmacol 83: 122-128. doi:10.1124/mol.112.080689. PubMed: 23066090. 23066090 PMC3533471

[39] Arthur-Farraj P, Wanek K, Hantke J, Davis CM, Jayakar A et al. (2011) Mouse schwann cells need both NRG1 and cyclic AMP to myelinate. Glia 59: 720-733. doi:10.1002/glia.21144. PubMed: 21322058. 21322058 PMC5722196

[40] Crawford AT, Desai D, Gokina P, Basak S, Kim HA (2008) E-cadherin expression in postnatal Schwann cells is regulated by the cAMP-dependent protein kinase a pathway. Glia 56: 1637-1647. doi:10.1002/glia.20716. PubMed: 18551621. 18551621 PMC2575062

[41] Howe DG, McCarthy KD (2000) Retroviral inhibition of cAMP-dependent protein kinase inhibits myelination but not Schwann cell mitosis stimulated by interaction with neurons. J Neurosci 20: 3513-3521. PubMed: 10804191. 10804191 10.1523/JNEUROSCI.20-10-03513.2000 PMC6772664

[42] Houslay MD (2010) Underpinning compartmentalised cAMP signalling through targeted cAMP breakdown. Trends Biochem Sci 35: 91-100. doi:10.1016/j.tibs.2009.09.007. PubMed: 19864144. 19864144

[43] Gerdin MJ, Eiden LE (2007) Regulation of PC12 cell differentiation by cAMP signaling to ERK independent of PKA: do all the connections add up? Sci STKE 2007: pe15. 10.1126/stke.3822007pe15 PMC4183209 17440132

[44] Kiermayer S, Biondi RM, Imig J, Plotz G, Haupenthal J et al. (2005) Epac activation converts cAMP from a proliferative into a differentiation signal in PC12 cells. Mol Biol Cell 16: 5639-5648. doi:10.1091/mbc.E05-05-0432. PubMed: 16207818. 16207818 PMC1289409

[45] Ravni A, Vaudry D, Gerdin MJ, Eiden MV, Falluel-Morel A et al. (2008) A cAMP-dependent, protein kinase A-independent signaling pathway mediating neuritogenesis through Egr1 in PC12 cells. Mol Pharmacol 73: 1688-1708. doi:10.1124/mol.107.044792. PubMed: 18362103. 18362103 PMC4188547

[46] Parkinson DB, Dickinson S, Bhaskaran A, Kinsella MT, Brophy PJ et al. (2003) Regulation of the myelin gene periaxin provides evidence for Krox-20-independent myelin-related signalling in Schwann cells. Mol Cell Neurosci 23: 13-27. doi:10.1016/S1044-7431(03)00024-1. PubMed: 12799134. 12799134

[47] Iyengar R (1996) Gating by cyclic AMP: expanded role for an old signaling pathway. Science 271: 461-463. doi:10.1126/science.271.5248.461. PubMed: 8560257. 8560257

[48] Morrissey TK, Levi AD, Nuijens A, Sliwkowski MX, Bunge RP (1995) Axon-induced mitogenesis of human Schwann cells involves heregulin and p185erbB2. Proc Natl Acad Sci U S A 92: 1431-1435. doi:10.1073/pnas.92.5.1431. PubMed: 7877996. 7877996 PMC42533

[49] Lyons DA, Pogoda HM, Voas MG, Woods IG, Diamond B et al. (2005) erbb3 and erbb2 are essential for schwann cell migration and myelination in zebrafish. Curr Biol 15: 513-524. doi:10.1016/j.cub.2005.02.030. PubMed: 15797019. 15797019

[50] Birchmeier C, Nave KA (2008) Neuregulin-1, a key axonal signal that drives Schwann cell growth and differentiation. Glia 56: 1491-1497. doi:10.1002/glia.20753. PubMed: 18803318. 18803318

[51] Monk KR, Naylor SG, Glenn TD, Mercurio S, Perlin JR et al. (2009) A G protein-coupled receptor is essential for Schwann cells to initiate myelination. Science 325: 1402-1405. doi:10.1126/science.1173474. PubMed: 19745155. 19745155 PMC2856697

[52] Monk KR, Oshima K, Jörs S, Heller S, Talbot WS (2011) Gpr126 is essential for peripheral nerve development and myelination in mammals. Development 138: 2673-2680. doi:10.1242/dev.062224. PubMed: 21613327. 21613327 PMC3109596

[53] Leitman EM, Tewari A, Horn M, Urbanski M, Damanakis E et al. (2011) MLCK regulates Schwann cell cytoskeletal organization, differentiation and myelination. J Cell Sci 124: 3784-3796. doi:10.1242/jcs.080200. PubMed: 22100921. 22100921 PMC3225267

[54] Guo L, Moon C, Niehaus K, Zheng Y, Ratner N (2012) Rac1 controls Schwann cell myelination through cAMP and NF2/merlin. J Neurosci 32: 17251-17261. doi:10.1523/JNEUROSCI.2461-12.2012. PubMed: 23197717. 23197717 PMC3601465

