# Correction: Opposing Roles of pka and epac in the cAMP-Dependent Regulation of Schwann Cell Proliferation and Differentiation

**DOI:** 10.1371/annotation/fa651e8d-ed5a-4937-8d7e-8009b10dcbe0

**Published:** 2014-01-06

**Authors:** Ketty Bacallao, Paula V. Monje

The terms "PKA" and "EPAC" are not appropriately capitalized in the article title. The correct title is: 

"Opposing Roles of PKA and EPAC in the cAMP-Dependent Regulation of Schwann Cell Proliferation and Differentiation." 

The correct Citation is: 

Bacallao K, Monje PV (2013) Opposing Roles of pka and epac in the cAMP-Dependent Regulation of Schwann Cell Proliferation and Differentiation. PLoS ONE 8(12): e82354. doi:10.1371/journal.pone.0082354.

